# Isolation and Characterization of Collagen and Antioxidant Collagen Peptides from Scales of Croceine Croaker (*Pseudosciaena crocea*)

**DOI:** 10.3390/md11114641

**Published:** 2013-11-21

**Authors:** Bin Wang, Yu-Mei Wang, Chang-Feng Chi, Hong-Yu Luo, Shang-Gui Deng, Jian-Yin Ma

**Affiliations:** 1 School of Food and Pharmacy, Zhejiang Ocean University, 1 Ocean University S.Rd, Lincheng New District, Zhoushan 316022, China; E-Mails: wangym731@126.com (Y.-M.W.); hyluo11@hotmail.com (H.-Y.L.); dengshanggui@163.com (S.-G.D.); chymjy@zjou.edu.cn (J.-Y.M.); 2 National Engineering Research Center of Marine Facilities Aquaculture, School of Marine Science and Technology, Zhejiang Ocean University, 1 Ocean University S.Rd, Lincheng New District, Zhoushan 316022, China; E-Mail: chichangfeng@hotmail.com

**Keywords:** croceine croaker (*Pseudosciaena crocea*), scale, acid soluble collagen (ASC), peptide, antioxidant activity

## Abstract

Acid soluble collagen (ASC) from scales of croceine croaker (ASC-C) was successfully isolated with the yield of 0.37% ± 0.08% (dry weight basis), and characterized as type I collagen on the basis of amino acid analysis and electrophoretic pattern. The antioxidant hydrolysate of ASC-C (ACH) was prepared through a two-stage *in vitro* digestion (4-h trypsin followed by 4-h pepsin), and three antioxidant peptides (ACH-P1, ACH-P2, and ACH-P3) were further isolated from ACH using ultrafiltration, gel chromatography, and RP-HPLC, and their amino acid sequences were identified as GFRGTIGLVG (ACH-P1), GPAGPAG (ACH-P2), and GFPSG (ACH-P3). ACH-P1, ACH-P2, and ACH-P3 showed good scavenging activities on hydroxyl radical (IC_50_ 0.293, 0.240, and 0.107 mg/mL, respectively), DPPH radical (IC_50_ 1.271, 0.675, and 0.283 mg/mL, respectively), superoxide radical (IC_50_ 0.463, 0.099, and 0.151 mg/mL, respectively), and ABTS radical (IC_50_ 0.421, 0.309, and 0.210 mg/mL, respectively). ACH-P3 was also effectively against lipid peroxidation in the model system. The antioxidant activities of three collagen peptides were due to the presence of hydrophobic amino acid residues within the peptide sequences. The collagen peptides might be used as antioxidant for the therapy of diseases associated with oxidative stress, or reducing oxidative changes during storage.

## 1. Introduction

Oxidation of biomolecules has been identified as a free radical mediated process, which results in numerous unfavorable impacts on food and biological systems [1]. In aerobic organisms, reactive oxygen species (ROS) including superoxide anion radical, hydroxyl radical, hydrogen peroxide, singlet oxygen, and hypochlorous acid, are produced inside the cell under physiological and pathological conditions in response to external stimuli and chemicals [2]. Uncontrolled generation of free radicals causes havoc in biological system by damaging all major groups of biomolecules (DNA, protein, lipids and small cellular molecules), which in turn leads to cardiovascular and neurodegenerative diseases [3]. In foods, development of rancid flavor and undesirable chemical compounds are the result of free radical mediated oxidation of fatty acids and lipids. Furthermore, oxidation of food lipids leads to the deterioration of food quality and shortens the shelf life [4]. Hence, antioxidants play a vital role in both food systems as well as in the human body to reduce oxidative processes. At present, butylated hydroxytoluene (BHT) and butylated hydroxyanisole (BHA) are extensively used as antioxidants in order to reduce the damage caused by free radicals. However, the possible toxicity as well as general consumer rejection leads to decreasing use of these synthetic antioxidants [5]. Therefore, there is a growing interest in identifying antioxidants from natural sources including some dietary protein compounds.

It was reported that proteins possessed significant antioxidant activities because they could inactivate ROS, scavenge free radicals, chelate prooxidative transition metals, reduce hydroperoxides, and enzymatically eliminate specific oxidants [6]. Certain amino acid residues and their specific sequences are thought to be responsible for the activities [6,7,8]. Collagen is the main protein of connective tissue in animals and the most abundant protein in mammals, constituting approximately 30% of total protein in animal body [9,10]. At present, collagen and collagen peptides have been widely utilized as a material for food additives, cosmetics, biomedical materials and pharmaceuticals due to their excellent biocompatibilites and biodegradabilities, and weak antigenicities [11]. Recently, fish by-products, such as skins, bones, scales and fins, were used as alternatives of the skins of land-based animals for preparing collagens because of their high availabilities and yields of collagen, and absence of risk disease transmission and religious barriers [12,13,14,15,16,17,18,19], and some peptides including EGL, YGDEY, GPHypGPHypGPHypGPHypGPHypG, FDSGPAGVL, NGPLQAGQPGER, and HGPLGPL were released by enzymatic proteolysis of collagen and showed high antioxidant acitivities [6,20,21], which gave us more information on by-product reuse. Hence, our attention was focused on the identification and characterization of antioxidant collagen peptides from fish by-products.

Approximately 49,000 tons of scales per year, constituting about two percent of fish weight, are generated during fish de-scaling processing. Fish scales are composed of connective tissue protein, collagen, covered with calcium salts. The amount of protein ranges from 41% to 84% and the remaining is calcium phosphate and calcium carbonate [22]. So far, many papers have reported the biochemical properties of collagens extracted from scales of carp [23], sardine [24], red sea bream [24], Japanese sea bass [24], snakehead [25], spotted golden goatfish [26], bighead carp [10], grass carp [27], black drum [27], sheepshead seabream [27], red seabream [28], Nile tilapia [28], and barramundi [29]. These collagens are mainly type I collagen with higher denaturation temperatures and imino acid contents than fish skin collagen, and more suitable as the alternative sources of porcine dermis collagens. However, no information regarding collagen and collagen peptides from scales of croceine croaker (*Pseudosciaena crocea*) has been reported. Thus, the objectives of this paper are to isolate and characterize collagen and collagen peptides from scales of croceine croaker, and to evaluate antioxidant properties of collagen peptides using radical scavenging assays and lipid peroxidation inhibition assay.

## 2. Results and Discussion

### 2.1. Characterization of Collagen

#### 2.1.1. Proximate Analysis

Scales of croceine croaker contained ash (46.73 ± 2.50 g/100 g) as a major component, followed by moisture (25.71 ± 0.24 g/100 g), collagen (20.33 ± 0.93 g/100 g) and fat (7.04 ± 0.38 g/100 g), respectively. High ash content was found in the scales, which was mainly due to the presence of calcium-deficient hydroxyappatite (Ca_5_(PO_4_)_3_OH) localized in the upper osseous layer and lower fibrillar plate of scale. The yield of ASC-C was 0.37% ± 0.08% (dry weight basis), or 0.27% ± 0.06% (wet weight basis), which was similar to that of ASC from spotted golden goatfish scales (0.46% on the basis of dry weight) [26], but significantly lower than that of ASC from carp scales (0.86% on the basis of dry weight) [22]. ASC-C possessed high protein content (93.56 ± 1.80 g/100 g), low moisture content (4.52 ± 0.54 g/100 g), and trace amounts of ash (1.03 ± 0.54 g/100 g) and fat (0.43 ± 0.15 g).

#### 2.1.2. Amino Acid Composition

Amino acid compositions of ASC-C and type I collagen from calf skin (CSC) were shown in Table 1. ASC-C had Gly as the major amino acid (347.1 residues/1000 residues) and was rich in Ala (124.5 residues/1000 residues), Pro (110.0 residues/1000 residues) and Hyp (79.4 residues/1000 residues). Low contents of His (8.2 residues/1000 residues), Hyl (5.5 residues/1000 residues), Tyr (4.1 residues/1000 residues) and Cys (3.2 residue/1000 residues) were also observed, but Trp were not detected. The result indicated that the experiment of removing non-collagenous proteins was appropriate. According to the literatures [9,13], Gly is the most dominant amino acid in collagen, and all members of the collagen family are characterized by domains with repetitions of the proline-rich tripeptides (Gly-X-Y) involved in the formation of the triple helix, except for the first 14 amino acid residues from the *N*-terminus and the first 10 amino acid residues from the *C*-terminus of the collagen molecules, where X is generally Pro and Y is mainly Hyp. On the other hand, the Gly content of ASC-C (347.1 residues/1000 residues) was higher than those (328–341 residues/1000 residues) of ASC from scales of carp [23], deep-sea redfish [30], sardine [24], red sea bream [24], Japanese sea bass [24], and spotted golden goatfish [26], but lower than those of ASC from rohu (361 residues/1000 residues) and catla (353 residues/1000 residues) scales [31].

**Table 1:** Amino acid compositions of ASC-C and CSC (residues/1000 residues).

Amino Acid	ASC-C	CSC	Amino Acid	ASC-C	CSC
Hyp	79.4	95.1	Leu	23.9	23.4
Asp	40.7	45.7	Tyr	4.1	3.7
Thr	24.2	18.4	Phe	15.2	3.3
Ser	30.5	33.2	Hyl	5.5	7.7
Glu	64.7	75.9	Lys	24.8	26.5
Pro	110.0	121.5	His	8.2	5.3
Gly	347.1	330.6	Arg	44.9	51.0
Ala	124.5	119.7	Met	13.2	6.1
Cys	3.2	0.0	Trp	0.0	0.0
Val	24.5	21.5	Imino acid	189.4	216.6
Ile	11.4	11.4	Total	1000.0	1000.0

Additionally, the amounts of imino acid (Pro and Hyp) are important for the structural integrity of collagen. The imino acid content of ASC-C was 189.4 residues/1000 residues, which was similar to those of ASC from scales of carp (192 residues/1000 residues) [23], Japanese sea bass (193 residues/1000 residues) [24], red sea bream (196 residues/1000 residues) [24], sardine (197 residues/1000 residues) [24], and spotted golden goatfish (196 residues/1000 residues) [26], lower than those of ASC from scales of rohu (201 residues/1000 residues) and catla (214 residues/1000 residues).

It was reported that pyrrolidine rings of imino acid (Pro and Hyp) imposed restrictions on the conformation of the polypeptide chain and helped to strengthen thermal stability of the triple helix [14,32]. In particular, Hyp is believed to play a key role in the stabilization of the triple-stranded collagen helix due to its hydrogen bonding ability through its hydroxyl group. Therefore, the helices of ASC-C might be less stable than that of CSC (216.6 residues/1000 residues) due to the lower imino acid content.

#### 2.1.3. Electrophoretic Pattern of ASC-C

Apart from amino acid composition, the properties of collagen are also influenced by the distribution of the molecular weights and composition of its subunits.

**Figure 1:**
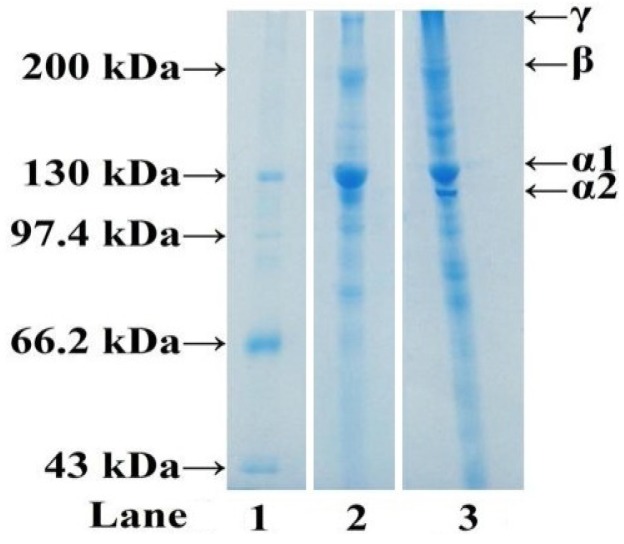
SDS-PAGE pattern ASC. Lane 1, protein markers; lane 2, ASC; lane 3, CSC.

SDS-PAGE patterns of ASC-C and CSC analyzed by 7.5% separating gel were shown in Figure 1. Similar protein patterns were observed from ASC-C and CSC. ASC-C consisted of two α-chains, and the band intensities of α1-chains were approximately two-fold higher than those of α2-chains. High molecular weight components, particularly β (dimmers) and γ (trimers) components, as well as other cross-linked molecules with higher molecular weight were also observed. The result suggested that ASC-C were type I collagen, which was accordance with ASC from scales of snakehead fish [25], sardine [24], red seabream [24], Japanese seabass [24], rohu and catla [31] and spotted golden goatfish [26]. It is generally known that type I collagen consists of two α1- and one α2-chain as the major component ([α1]2α2). However, it was reported that another heterotrimer of type I collagen (α1α2α3) was found as a major component in ASC from the scales of sheephead and black drum [27]. The α3 chain might be present in ASC-C because it had a similar MW to α1-chain and could not be separated from the α1-chain using SDS-PAGE.

### 2.2. Isolation of Antioxidant Peptides from ASC-C

#### 2.2.1. Double-Enzyme Hydrolysis and Preparation of Hydrolysate of ASC-C (ACH)

According to the literature [33], hydrolysis using endogenous enzymes, such as trypsin, chymotrypsin, pepsin, other enzymes of the viscera and digestive tract, as well as lysosomal proteases or catheptic enzymes in fish or other invertebrate muscle cells, has been the main process for producing antioxidative peptides from fish proteins. Careful choice of a suitable enzyme and digestion conditions, such as temperature and pH, for the optimal activity of enzyme, as well as the control of hydrolysis time and enzyme-to-substrate ratio, are crucial for obtaining protein hydrolysates with desirable functional and bioactive properties. In our previous research, trypsin hydrolysates of cartilage collagens [34] showed good radical scavenging activity. In addition, collagen from scales of croceine croaker was easily soluble at acidic pH (1–4), and the optimal pH of pepsin was 2, which might help the interaction between pepsin and collagen. So, trypsin and pepsin were chosen in the hydrolysis of collagen from scales of croceine croaker.

Using DH (%) and hydroxyl radical scavenging activity (%) as targets, the enzymatic conditions of ACH for pepsin (enzymolysis time 4 h, enzyme-to-substrate ratio 2.5%, enzymolysis temperature 37 °C and pH 2) and trypsin (enzymolysis time 4 h, enzyme-to-substrate ratio 3.0%, enzymolysis temperature 40 °C and pH 8.0) were optimized by single-factor test and orthogonal experiment (data not shown), and double enzyme (trypsin + pepsin) hydrolysis of ASC-C was further applied (Table 2).

As shown in Table 2 below, DE-3 (ASC-C was hydrolyzed with trypsin at its optimum then pepsin at its optimum) had the highest DH and hydroxyl radical scavenging activity among four double-enzyme hydrolysis treatments. The result was accordance with the previous reports that high DH and low molecular weight of hydrolysates made a great contribute to their antioxidant activities including DPPH/hydroxyl radicals scavenging activities and reducing power [5,35]. So, DE-3 was chosen to prepare the ASC-C hydrolysates (ACH).

**Table 2:** The degree of hydrolysis (DH) and hydroxyl radical scavenging activities of the hydrolysates from different enzymatic treatments.

Hydrolysates	DH (%)	Hydroxyl Radical Scavenging Activity (%)(*c* = 10 mg/mL)
**Trypsin**	17.47 ± 0.63	53.11 ± 0.97
**Pepsin**	13.73 ± 0.83	44.96 ± 1.97
**DE-1**	20.28 ± 1.05	62.33 ± 3.01
**DE-2**	19.67 ± 0.68	60.24 ± 3.38
**DE-3**	25.11 ± 0.67	85.92 ± 3.84
**DE-4**	22.61 ± 0.74	65.46 ± 2.56

**DE-1**: ASC-C was hydrolyzed with the mixture of trypsin and pepsin at the optimum of trypsin; **DE-2**: ASC-C was hydrolyzed with the mixture of trypsin and pepsin at the optimum of pepsin; **DE-3**: ASC-C was hydrolyzed with trypsin at its optimum then pepsin at its optimum; **DE-4**: ASC-C was hydrolyzed with pepsin at its optimum then trypsin at its optimum.

#### 2.2.2. Fractionation of ACH by Ultrafiltration

In order to obtain a fraction with the desired antioxidant activity, protein hydrolysate often was further processed through ultrafiltration membranes. So, low molecular mass membrane cut-offs were used for concentrating antioxidative peptides from the higher molecular mass components remaining, including undigested polypeptide chains and enzymes.

**Figure 2:**
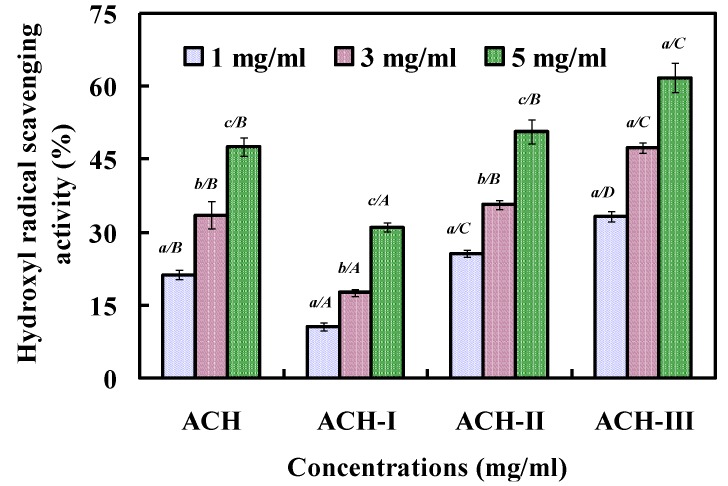
Hydroxyl radical scavenging activity of ACH and its three fractions by ultrafiltration. All the results were triplicates of mean ± SD; SD: Standard deviation. (*a*–*c*) Values with same letters indicated no significant difference of same sample at different concentrations (*p* > 0.05). (*A–D*) Values with same letters indicated no significant difference of different sample at same concentrations (*p* > 0.05).

In the test, ACH solution was filtered using two ultrafiltration membranes with 1 and 3 kDa molecular-weight-cut-offs to obtain three fractions corresponding to molecular weights (MW) above 3 kDa (ACH-I), between 1 and 3 kDa (ACH-II), and below 1 kDa (ACH-III). As shown in Figure 2, ACH, ACH-I, ACH-II, and ACH-III scavenged hydroxyl radical in a concentration dependent way with EC_50_ values of 5.16, 8.67, 5.70, and 3.35 mg/mL, respectively. ACH-III showed higher antioxidant activity than the other two fractions at the tested concentrations, and there were significant difference on antioxidant activity between ACH-III and other sample at the same concentrations, Ranathunga, Rajapakse, & Kim (2006) reported that the antioxidant activity of hydrolysates depended on their molecular weight distributions, and hydrolysates or peptides with lower molecular weight could be easier to cross the intestinal barrier to exert their biological effects, and interact more effectively with free radicals interfering in the oxidative process [36]. So, the higher antioxidant activity of ACH-III was due to its low molecular weight.

#### 2.2.3. Gel Filtration Chromatography of ACH-III

**Figure 3:**
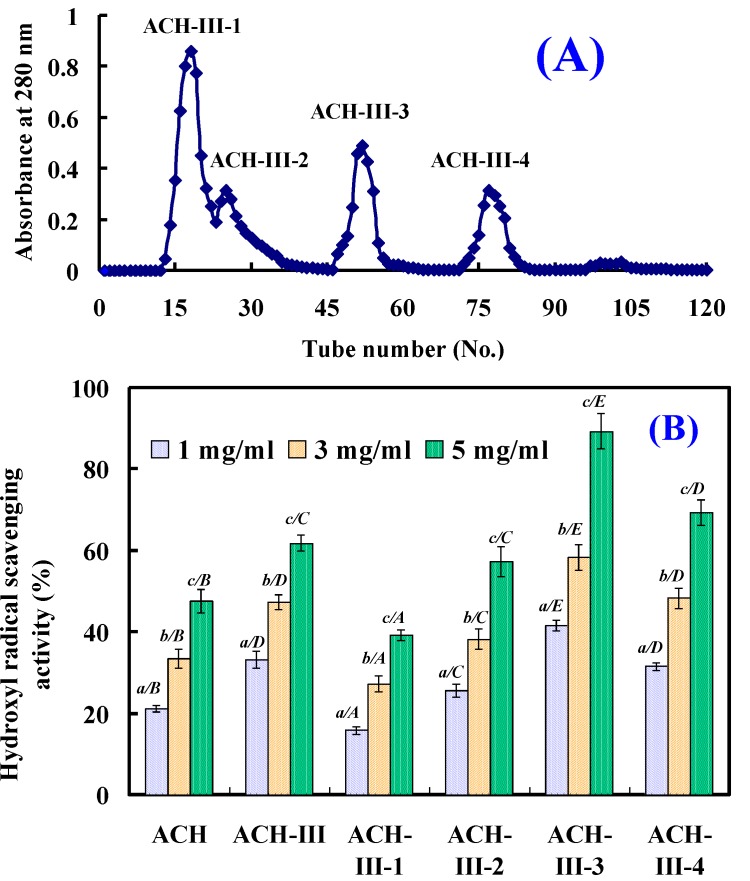
Gel filtration chromatography of ACH-III on a Sephadex G-15 column (**A**) and hydroxyl radical scavenging activity of its four subfractions (**B**). All the results were triplicates of mean ± SD; SD: Standard deviation. (*a*–*c*) Values with same letters indicated no significant difference of same sample at different concentrations (*p* > 0.05). (*A*–*E*) Values with same letters indicated no significant difference of different sample at same concentrations (*p* > 0.05).

The lyophilized ACH-III was further purified by size exclusion chromatography on Sephadex G-15 column, and was separated into four subfractions (ACH-III-1, ACH-III-2, ACH-III-3, and ACH-III-4) (Figure 3). Each subfraction was pooled and lyophilized, and its antioxidant activity was assayed. As shown in Figure 3, ACH-III-3 showed the highest hydroxyl radical scavenging activity among the four subfractions with EC_50_ values of 1.90 mg/mL, which was lower than the EC_50_ values of ACH (EC_50_ 5.16 mg/mL), ACH-III (EC_50_ 3.35 mg/mL), and ACH-III-1 (EC_50_ 8.91 mg/mL), ACH-III-2 (EC_50_ 4.21 mg/mL), and ACH-III-4 (EC_50_ 3.03 mg/mL), respectively. Antioxidant activity of ACH-III-3 showed significant difference to other tested sample at the same concentrations.

#### 2.2.4. Isolation Peptides from ACH-III-3 by RP-HPLC

**Figure 4:**
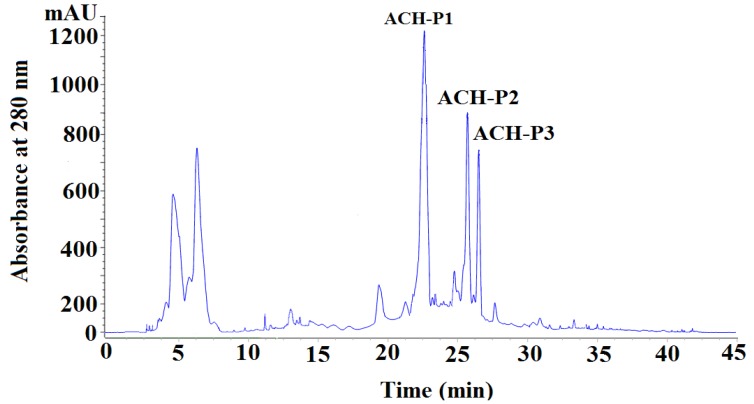
Chromatography of ACH-III-3 separated by RP-HPLC. Elution was performed with the 30% acetonitrile containing 0.1% TFA and monitored at 280 nm.

The subfraction of ACH-III-3 was further separated by RP-HPLC using 30% acetonitrile containing 0.1% trifluoroacetic acid (TFA). The elution profile of peptides detected at 280 nm was shown in Figure 4, and three purified peptides named as ACH-P1, ACH-P2, and ACH-P3 were isolated, collected, and lyophilized.

### 2.3. Amino Acid Sequence Analysis and Molecular Mass Determination

The N-terminal amino acid sequences and molecular mass of ACH-P1, ACH-P2, and ACH-P3 were determined using Protein/Peptide Sequencer and ESI-MS, respectively. Accordingly, the amino acid sequence of ACH-P1 was determined to be Gly-Phe-Arg-Gly-Thr-Ile-Gly-Leu-Val-Gly (GFRGTIGLVG), and the detected molecular mass (976.5536 Da) agreed well with the theoretical mass calculated from the sequence. The amino acid sequence of ACH-P2 was determined to be Gly-Pro-Ala-Gly-Pro-Ala-Gly (GPAGPAG), and the detected molecular mass (526.2456 Da) agreed well with the theoretical mass calculated from the sequence. The amino acid sequence of ACH-P3 was determined to be Gly-Phe-Pro-Ser-Gly (GFPSG) and the detected molecular mass (463.4070 Da) agreed well with the theoretical mass calculated from the sequence (Supplementary Figures S1–S3).

### 2.4. Antioxidative Activity of ACH-P1, ACH-P2, and ACH-P3

**Figure 5:**
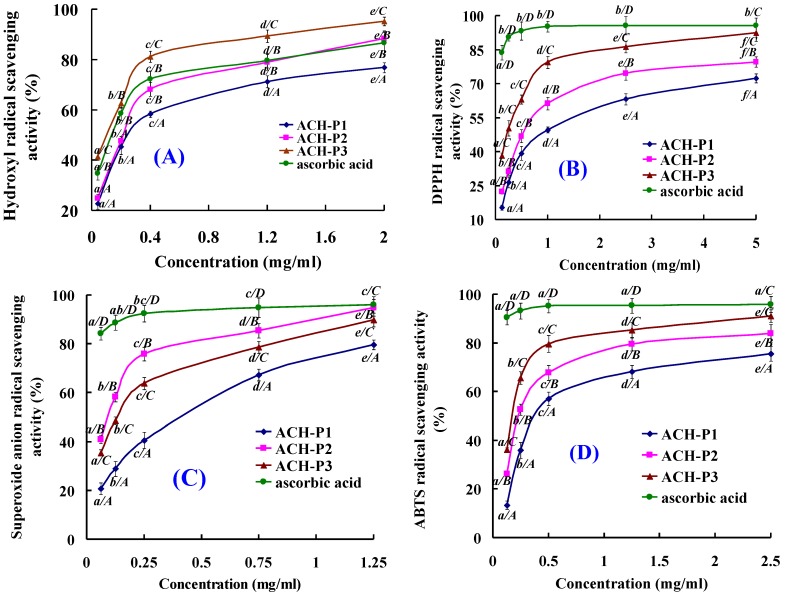
Hydroxyl radical (**A**), DPPH radical (**B**), superoxide radical (**C**), and ABTS radical (**D**) scavenging activities of ACH-P1, ACH-P2, and AC-P3. All the values were mean ± SD; SD: Standard deviation. (*a*–*e*) Values with same letters indicated no significant difference of same sample at different concentrations (*p* > 0.05). (*A*–*D*) Values with same letters indicated no significant difference of different sample at same concentrations (*p* > 0.05).

Due to the complexity of oxidative processes occurring in food or biological systems as well as the different antioxidant mechanisms by which various compounds may act, finding one method that can evaluate the antioxidant activity of compounds is not an easy task. Therefore, methods such as the oxygen radical absorbance capacity (ORAC) assay, Trolox equivalent antioxidant capacity (TEAC) assay, and the total radical-trapping antioxidant parameter (TRAP) assay have been widely reported in the literature for measuring antioxidant capacity of food and biological samples [8]. In the test, four kinds of radical scavenging assays and lipid peroxidation inhibition assay were employed to evaluate the antioxidant activities of ACH-P1, ACH-P2, and ACH-P3 (Figure 5).

#### 2.4.1. Hydroxyl Radical Scavenging Activity

As shown in Figure 5A ACH-P1 ACH-P2 and ACH-P3 scavenged hydroxyl radicals in a concentration dependent way, and ACH-P3 exhibited stronger scavenging activity on hydroxyl radicals at all the tested concentrations. IC_50_ of ACH-P1, ACH-P2, and ACH-P3 were 0.293, 0.240, and 0.107 mg/mL, respectively, which were significantly higher than ACH (IC_50_ 5.16 mg/mL), ACH-III (IC_50_ 3.35 mg/mL), and ACH-III-3 (IC_50_ 1.90 mg/mL). As reported in the literatures [37,38,39,40,41], some antioxidant peptides were isolated from other aquatic products and their by-products. The IC_50_ of ACH-P3 was higher than those of PSYV (2.64 mg/mL) from loach protein [37], YPPAK (0.228 mg/mL) from blue mussel [38], WDR (0.15 mg/mL) and PYFNK (0.24 mg/mL) from ethanol-soluble protein hydrolysate of scalloped hammerhead [8], NGPLQAGQPGER (0.123 mg/mL), NGLEGLK (0.313 mg/mL) and NADFGLNGLEGLA (0.612 mg/mL) from giant squid protein hydrolysate [24,39], LKQELEDLLEKQE (0.046 mg/mL) from oyster protein hydrolysate [40], but only lower than those of FDSGPAGVL (0.078 mg/mL) from giant squid protein hydrolysate [21], MQIFVKTLTG (0.005 mg/mL), and DLSDGEQGVL (0.007 mg/mL) from venison protein hydrolysate [41]. So, three antioxidant pentapeptides, especially ACH-P3, showed high activities against hydroxyl radicals, which indicated that they could be used as the scavenging agents for protecting hydroxyl radical-induced damage in the living body.

#### 2.4.2. DPPH Radical Scavenging Activity

DPPH radical scavenging assay is quick, convenient, and efficient in predicting the antioxidant activities of protein hydrolysates, their fractions, and purified peptides [42]. Therefore, the relatively stable DPPH radical has been widely used to test the ability of compounds to act as free radical scavengers or hydrogen donors and thus to evaluate the antioxidant activity. As shown in Figure 5B, ACH-P1, ACH-P2, and ACH-P3 showed concentration-dependent, anti-DPPH radical activity with IC_50_ of 1.271, 0.675, and 0.283 mg/mL, respectively. ACH-P3 showed highest DPPH radical scavenging activity among the proteins hydrolysate, fractions, and purified peptides. The IC_50_ of ACH-P1, ACH-P2, and ACH-P3 were higher than those of PSYV (17.0 mg/mL) from loach protein [37], YPPAK (2.62 mg/mL) from blue mussel [38], WDR (3.63 mg/mL) and PYFNK (4.11 mg/mL) from ethanol-soluble protein hydrolysate of scalloped hammerhead [8]. The result suggested that three collagen peptides from croceine croaker scales could react with DPPH free radicals to convert them to less harmful or unharmful products and break the radical chain reaction. However, scavenging activity of AA, a known antioxidant used as positive control, was relatively more pronounced than those of three collagen peptides.

#### 2.4.3. Superoxide Anion Radical Scavenging Activity

Superoxide radical can promote oxidative reaction due to its ability in reducing transition metals, and it can release protein-bound metals and form perhydroxyl radicals which initiate lipid oxidation. For cytoprotection against this reactive oxygen, superoxide dismutase (SOD), which catalyzes the neutralization of superoxide anion to hydrogen peroxide, is one of the defense mechanisms in the living cell. The scavenging effect of three antioxidant peptides on superoxide radicals was investigated at different concentrations (0.0625–1.25 mg/mL), and the antioxidant activity drastically increased with increasing concentration of the peptide (Figure 5C). ACH-P1, ACH-P2, and ACH-P3 could effectively scavenge superoxide radicals with IC_50_ value of 0.463, 0.099, and 0.151 mg/mL, and ACH-P2 showed strongest scavenging activity on superoxide radicals at all the tested concentrations. The IC_50_ of ACH-P2 was higher than those of LKQELEDLLEKQE (IC_50_ 0.128 mg/mL) from oyster protein hydrolysate [40], NADFGLNGLEGLA (IC_50_ 0.864 mg/mL) and NGLEGLK (IC_50_ 0.419 mg/mL) from giant squid muscle protein hydrolysate [39], and PYFNK (IC_50_ 0.11 mg/mL) from ethanol-soluble protein hydrolysate of scalloped hammerhead [8], but lower than those of WDR (IC_50_ 0.09 mg/mL) from ethanol-soluble protein hydrolysate of scalloped hammerhead [8], YPPAK (0.072 mg/mL) from blue mussel [38], YFYPEL (IC_50_ 0.066 mg/mL) from casein protein hydrolysate [43], VECYGPNRPQF (IC_50_ 0.010 mg/mL) from algae protein hydrolysate [44]. Compared with those reported peptides, ACH-P2 showed strong superoxide radical scavenging activity, and it was presumed that the purified peptide might have high SOD-like activity, thereby scavenging superoxide radicals in living cells.

#### 2.4.4. ABTS Radical Scavenging Activity

ABTS radical assay is an excellent tool for determining the antioxidative activity, in which the radical is quenched to form ABTS radical complex. Meanwhile, it is more sensitive to determine antioxidative capacities of protein hydrolysates samples, because it can determine their capacities at lower inhibition concentrations. With increasing concentration, all peptides showed increased ABTS radical scavenging activities (Figure 5D). At the same concentration, ACH-P3 showed the highest activity. The IC_50_ values of ACH-P1, ACH-P2, and ACH-P3 were 0.421, 0.309, and 0.210 mg/mL. For other reports, WDR (IC_50_ 0.34 mg/mL and 0.12 mg/mL) and PYFNK (IC_50_ 0.12 mg/mL) from EP hydrolysate of scalloped hammerhead [8], and hydrolysate fractions (consisted of Trp-Pro-Leu, Val-Pro-Trp and Val-Phe-Pro-Trp) from buckwheat protein also showed high potential in scavenging ABTS radical. Therefore, the three peptides were able to scavenge ABTS radicals, leading to the termination of radical chain reaction, thereby preventing lipid oxidation via a chain breaking reaction.

#### 2.4.5. Lipid Peroxidation Inhibition Assay

Peroxidation of lipids is a complex process that involves formation and propagation of lipid radicals and lipid hydroperoxides in the presence of oxygen. Each assay, such as DPPH, hydroxyl, and superoxide radical scavenging, measured an antioxidant property representing a different mechanism, which could not reflect the multiple mechanisms by which sample acted as antioxidant to retard or inhibit lipid oxidation in a food system. Therefore, the activity of ACH-P1, ACH-P2, and ACH-P3 against the peroxidation of linoleic acid was investigated and compared to that of BHT in this section.

**Figure 6:**
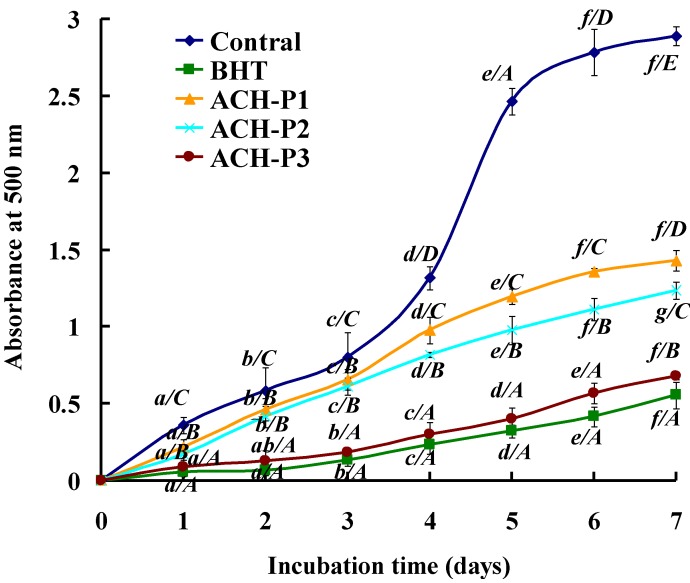
Lipid peroxidation inhibition of ACH-P1, ACH-P2, and ACH-P3. All the values were mean ± SD; SD: Standard deviation. (*a*–*e*) Values with same letters indicated no significant difference of the same sample at different concentrations (*p* > 0.05); (*A*–*E*) Values with same letters indicated no significant difference of different sample at same concentrations (*p* > 0.05).

As shown in Figure 6, the control (without antioxidant) had the highest absorbance value in the test, indicating the highest degree of oxidation among the samples after a seven-day incubation, whereas ACH-P1, ACH-P2, and ACH-P3 effectively inhibited lipid peroxidation in linoleic acid emulsion system up to the seven days, and the activity of ACH-P3 was similar to that of positive control (BHT). Tsuge *et al.* (1991) [45] and Chen *et al.* (1996) [7] reported high inhibitory activities for AHK and PHH, respectively, in the linoleic acid peroxidation system. Since most peptides are amphiphilic in nature, they have the ability to stay in the lipid-water interface and effectively scavenge radicals present in both aqueous as well as oil phase of these model systems. Antioxidative peptides are therefore promising candidates to reduce lipid peroxidation in emulsion-type food products, as well as potentially in the human membrane systems.

#### 2.4.6. Relationship between Antioxidant Activities and Amino Acid Compositions of Peptides

Enzymatic hydrolysis is one of the approaches for the effective release of antioxidant peptides from protein sources. During hydrolysis, a wide variety of smaller peptides are generated, depending on enzyme specificity and the hydrolysis time. Changes in size, level and composition of free amino acids affect the antioxidant activities of peptides [46].

In the test, ACH-P1, ACH-P2, and ACH-P3, especially ACH-P3, exhibited good antioxidant activity on radical scavenging assays and lipid peroxidation inhibition assay, and the result was in concert with the general finding that short peptides with 2–10 amino acids exerted greater antioxidant potential and other bioactive properties than their parent native proteins or large polypeptides [36].The finding was expected due to a higher possibility of smaller antioxidant molecules to interact more effectively with free radicals and inhibit the propagation cycles of lipid peroxidation.

The kind of amino acid residues within the peptide sequences was considered as one of the important factors for peptides’ activities. Gly and Pro have been suggested to play an important role in radical scavenging ability of some peptides. The side-chain of Gly consists of a single hydrogen atom and may confer high flexibility on the peptide backbone. The pyrrolidine ring of Pro tends to interrupt the secondary structure of the peptide imposing conformational constraints [40,46]. Zhang *et al.* (2009) reported that Phe and Gly have been shown to act positively as direct radical scavengers due to their ability to quench unpaired electrons or radicals by supporting protons [47]. Zhu *et al.* (2008) reported that the antioxidative potency of peptides containing Leu has been attributed to its long aliphatic side-chain group that is conceivably capable of interaction with acyl chains of susceptible fatty acids [48]. Moreover, in the sequence of ACH-P1, ACH-P2, and ACH-P3, there are four, two, and three hydrophobic amino acids, representing 40%, 28.5%, and 60% of the peptide chain, respectively. Since hydrophobicity of antioxidants is important for accessibility to hydrophobic targets, the presence of hydrophobic amino acids in the three purified peptide sequence was thought to be critical for the lipid oxidation inhibition by increasing solubility of peptides in lipid. According to the literatures [40,47,48], it was assumed that the presence of Gly, Ile, Leu, and Val residues within the sequence of ACH-P1, both Gly and Pro within the sequence of ACH-P2, and Gly, Phe, and Pro within the sequence of ACH-P3 seemed to play a key role for their antioxidant activities. The hydrophobic amino acids might help the peptides for accessibility to hydrophobic targets and increasing the affinity and reactivity of peptides from the cell membrane in the living cells [21].

The present research indicated that ACH-P3 was very successful in this process, which makes it of great interest as an antioxidant.

## 3. Experimental Section

### 3.1. Materials

The frozen croceine croaker (*P. crocea*), with average body weight of 300–350 g, was obtained from Zhejiang Dahaiyang Sci-Tech Co., Ltd. in Zhejiang Province of China. To prepare scales for collagen extraction, the frozen fish samples were thawed with running tap water until the core temperature of fish reached 10 °C. The scales were manually removed with a filleting knife, washed with cold distilled water and kept at −20 °C prior to collagen extraction.

### 3.2. Preparation of Collagen from Scales

The collagens were extracted by the previous method with a slight modification [30]. All the preparation procedures were carried out at 4 °C. To remove non-collagenous proteins, the prepared scales were mixed with 0.1 M NaOH at a solid to alkali solution ratio of 1:10 (w/v) and continuously stirred for 6 h using a Magnetic Stirrer (IT-08/09, Shanghai Yiheng Technical Co., Ltd., Shanghai, China) at a speed of 300 rpm. The alkali solution was changed every 3 h. Then, the pretreated scales were washed with cold water until the neutral or faintly basic pH of wash water was obtained. The pretreated scales were further demineralized with 0.5 M EDTA-2Na (pH 7.4) using a solid to solution ratio of 1:10 (w/v) for 48 h with a continuous stirring, and the solution was changed every 12 h. Then, the demineralized scales were continuously stirred with 20 volumes of cold tap water for 10 min and the washing was performed for 3 times.

Pretreated scales were soaked in 0.5 M acetic acid with a solid to solvent ratio of 1:15 (w/v) for 48 h with a continuous stirring. The mixtures were filtered with two layers of cheesecloth. The collagen in the filtrate was precipitated by adding NaCl to a final concentration of 2.5 M in the presence of 0.05 M Tris (hydroxymethyl) aminomethane, pH 7.0. The resultant precipitate was collected by centrifugation at 20,000× *g* at 4 °C for 60 min using a CR21G refrigerated centrifuge (Hitachi, Ltd., Tokyo, Japan). The pellet was dissolved in a minimum volume of 0.5 M acetic acid and dialyzed against 25 volumes of 0.1 M acetic acid for 12 h. Thereafter, it was dialyzed against 25 volumes of distilled water for 48 h. The resulting dialysate was freeze-dried and referred to as “acid soluble collagen, ASC”, and ASC from scales of croceine croakers was referred to as ASC-C.

### 3.3. Characterization of Collagen

#### 3.3.1. Proximate Analysis

Moisture, ash, fat and protein contents of scales and ASC-C were determined according to the methods of AOAC (2003) with the method numbers of 950.46B, 920.153, 960.39 (a) and 928.08, respectively. The converting factor of 6.25 was used for calculation of protein content.

#### 3.3.2. Determination of Amino Acid Composition

In order to determine the amino acid composition, freeze-dried ASC-C was dissolved in distilled water to obtain a final concentration of 1 mg/mL, and an aliquot of 50 mL was dried and hydrolyzed in vacuum-sealed glass tubes at 110 °C for 24 h in the presence of constant boiling 6 mM HCl containing 0.1% phenol and using norleucine (Sigma-Aldrich, Inc., St. Louis, MO, USA) as the internal standard. After hydrolysis, samples were again vacuum-dried, dissolved in application buffer and injected into an automated amino acid analyser (HITACHI 835-50 Amino Acid Analyzer, Tokyo, Japan).

#### 3.3.3. SDS-polyacrylamide Gel Electrophoresis (SDS-PAGE) of ASC-C

Electrophoretic pattern of ASC-C was measured according to the method of Li *et al.* (2013) with a slight modification [49], using 7.5% separating gel and 4% stacking gel. The samples (10 μg proteins) were mixed with the sample loading buffer (60 mM Tris-HCl, pH 8.0, containing 25% glycerol, 2% SDS, and 0.1% bromophenol blue) at 4:1 (v/v) ratio in the presence of β-ME, then applied to sample wells and electrophoresed in an electrophoresis instrument (AE-6200, ATTO Corporation, Tokyo, Japan). The electrophoresis was carried out for about 4 h at a constant voltage of 100 V. After electrophoresis, the gel was fixed with 50% (v/v) methanol and 10% acetic acid for 30 min. The gel was then stained for 3 h with 0.05% (w/v) Coomassie blue R-250 in 15% (v/v) methanol and 5% (v/v) acetic acid. The gel was finally destained with 30% (v/v) methanol and 10% (v/v) acetic acid. High molecular weight markers (Shanghai Institute of Biochemistry, the Chinese Academy of Sciences, Shanghai, China) were used to estimate the molecular weight of proteins. Type I collagen from calf skins (CSC) (Sigma-Aldrich Co. LLC., Louis, IL, USA) was used as a standard.

### 3.4. Isolation of Antioxidant Peptides from ASC-C

#### 3.4.1. Double-Enzyme Hydrolysis and Preparation of Hydrolysate of ASC-C (ACH)

The ASC-C hydrolysate was prepared using two different enzymes (pepsin and trypsin), and the hydrolysis condition (enzyme-to-substrate ratio (E/S), hydrolysis temperature (T), pH and hydrolysis time (*t*)) of each enzyme was optimized using an orthogonal L_9_(4^3^) test design (data not shown). Double-enzyme hydrolysis was further applied to hydrolyze ASC-C. As shown in Table 2, the double enzyme hydrolysis included the progressive and mixed hydrolysis of two selected enzymes. The progressive hydrolysis is a single enzyme hydrolysis at its optimum, following the other enzyme hydrolysis at its optimum; the mixed hydrolysis is a mixture of double-enzyme hydrolysis at either optimum. After digestion, the hydrolysates were inactivated in boiling water for 15 min and centrifuged at 4500× *g* for 15 min. The supernatants were collected to measure their Degree of Hydrolysis (DH) and hydroxyl radical scavenging activities, and the hydrolysate with highest hydroxyl radical scavenging activity was referred to as ACH.

#### 3.4.2. Fractionation of ACH by Ultrafiltration

ACH solution was filtered using two ultrafiltration membranes with 1 and 3 kDa molecular-weight-cut-off to obtain three fractions corresponding to molecular weights (MW) above 3 kDa (ACH-I), between 1 and 3 kDa (ACH-II), and below 1 kDa (ACH-III). Three fractions were dialyzed, concentrated, and lyophilized.

#### 3.4.3. Gel Filtration Chromatography of ACH-III

100.0 mg of ACH-III was dissolved in 5 mL deionized water and purified by Sephadex G-15 gel filtration column (2.6 × 80 cm), which had been equilibrated previously with deionized water. The column was eluted with deionized water at a flow rate of 1 mL/min, and the elution solution was collected every 3 mL and four subfractions (ACH-III-1, ACH-III-2, ACH-III-3, and ACH-III-4) were collected and lyophilized.

#### 3.4.4. Isolation Peptides from ACH-III-3 by RP-HPLC

The subfraction of ACH-III-3 was further separated by reversed-phase high performance liquid chromatography (RP-HPLC, Agilent 1200 HPLC, Agilent Ltd., Santa Clara, CA, USA) on a Zorbax, SB C-18 column (column size: 4.6 × 250 mm, 5 μm particle size, Agilent, Santa Clara, CA, USA), using 30% acetonitrile containing 0.1% trifluoroacetic acid (TFA). The elution solution was detected at 280 nm and three purified peptides named as ACH-P1, ACH-P2, and ACH-P3 were isolated, collected, and lyophilized.

### 3.5. Characterization of Collagen Peptides

#### 3.5.1. Degree of Hydrolysis (DH)

DH analysis was performed according to the previously described method [50]. The hydrolysate (50 μL) was mixed with 0.5 mL of 0.2 M phosphate buffer, pH 8.2 and 0.5 mL of 0.05% TNBS reagent. TNBS was freshly prepared before use by diluting with DI water. The mixture was incubated at 50 °C for 1 h in a water bath. The reaction was stopped by adding 1 mL of 0.1 M HCl and incubating at room temperature for 30 min. The absorbance was monitored at 420 nm. L-Leucine was used as a standard. To determine the total amino acid content, mungbean meal was completely hydrolysed with 6 M HCl with a sample to acid ratio of 1:100 at 120 °C for 24 h. DH (%) was calculated using the following equation:

DH = [(A*_t_* − A_0_)/(A_max_ − A_0_)] × 100

where A*_t_* was the amount of a-amino acids released at time *t*, A_0_ was the amount of a-amino acids in the supernatant at 0 h, and A_max_ was the total amount of a-amino acids obtained after acid hydrolysis at 120 °C for 24 h.

#### 3.5.2. Amino Acid Sequence Analysis and Molecular Mass Determination

The antioxidant peptides of ACH-P1, ACH-P2, and ACH-P3 were subjected to *N*-terminal amino acid sequencing on an Applied Biosystems 494 protein sequencer (PerkinElmer Inc., Waltham, MA, USA). Edman degradation was performed according to the standard program supplied by Applied Biosystems. Accurate molecular masses of ACH-P1, ACH-P2, and ACH-P3 were determined using a Q-TOF micro™ Mass Spectrometer (Waters Inc., Milford, MA, USA) coupled with an electrospray ionization (ESI) source.

#### 3.5.3. Antioxidative Activity

In the test, four kinds of radical scavenging assays and lipid peroxidation inhibition assay were measured according to the method developed by Wang, Li, Chi, Zhang and Luo (2012) [8].

##### 3.5.3.1. Hydroxyl Radical Scavenging Activity

In this system, hydroxyl radicals are generated by the Fenton reaction. Hydroxyl radicals can oxidize Fe^2+^ into Fe^3+^, and only Fe^2+^ can combine with 1,10-phenanthroline to form a red compound (1,10-phenanthroline-Fe^2+^) with the maximum absorbance at 536 nm. The concentration of hydroxyl radical is reflected by the degree of decolorization of the reaction solution. Briefly, 1,10-phenanthroline solution (1.0 mL, 1.865 mM) and the sample (2.0 mL) were added into a screw-capped tube and mixed. The FeSO_4_·7H_2_O solution (1.0 mL 1.865 mM) was then pipetted into the mixture. The reaction was initiated by adding 1.0 mL H_2_O_2_ (0.03% v/v). After being incubated at 37 °C for 60 min in a water bath, the absorbance of the reaction mixture was measured at 536 nm against a reagent blank. The reaction mixture without any antioxidant was used as the negative control, and mixture without H_2_O_2_ was used as the blank. The hydroxyl radical scavenging activity (HRSA) was calculated by the following formula:

HRSA(%) = [(A_s_ − A_n_)/(A_b_ − A_n_)] × 100%

where A_s_, A_n_, and A_b_ were the absorbance values determined at 536 nm of the sample, the negative control, and the blank after reaction, respectively.

##### 3.5.3.2. DPPH Radical Scavenging Activity

Two milliliters of deionized water containing different concentrations of samples were placed in cuvettes, and then 500 μL of ethanol solution of DPPH (0.02%) and 1.0 mL of ethanol were added into. The control was conducted in the same manner, except that distilled water was used instead of sample. In blank, DPPH solution was substituted with ethanol. The antioxidant activity of the sample was evaluated by the inhibition percentage of DPPH radical with the following equation:

DPPH radical scavenging activity (%) = (A_0_ + A' − A)/A_0_ × 100%

where A was sample absorbance rate; A_0_ was the absorbance of control group; A' was the blank absorbance.

##### 3.5.3.3. Superoxide Anion Radical Scavenging Activity

In the experiment, superoxide anions were generated in 1 mL of nitrotetrazolium blue chloride (NBT) (2.52 mM), 1 mL of NADH (624 mM) and 1 mL of different concentrations of samples. The reaction was initiated by adding 1 mL of phenazine methosulfate (PMS) solution (120 μg) to the reaction mixture. The absorbance was measured at 560 nm against the corresponding blank after 5 min incubation at 25 °C. The capacity of scavenging the superoxide anion radical was calculated using the following equation:

Superoxide anions scavenging activity (%) = [(A_control_ − A_sample_)/A_control_] × 100%

where A_control_ was the absorbance without sample and A_sample_ was the absorbance with sample.

##### 3.5.3.4. ABTS Radical Scavenging Activity

The ABTS radical cation was generated by mixing ABTS stock solution (7 mM) with potassium persulphate (2.45 mM). Mixture was left in the dark at room temperature for 16 h. The ABTS radical solution was diluted in 5 mM phosphate buffered saline (PBS) pH 7.4, to an absorbance of 0.70 ± 0.02 at 734 nm. One milliliter of diluted ABTS radical solution was mixed with 1 mL of different concentrations of samples. Ten minutes later, the absorbance was measured at 734 nm against the corresponding blank. The ABTS scavenging activity of samples was calculated using the following equation:

ABTS scavenging activity (%) = [(A_control_ − A_sample_)/A_control_] × 100%

where A_control_ was the absorbance without sample and A_sample_ was the absorbance with sample.

##### 3.5.3.5. Lipid Peroxidation Inhibition Assay

A sample (5.0 mg) was dissolved in 10 mL of 50 mM phosphate buffer (pH 7.0), and added to a solution of 0.13 mL of linoleic acid and 10 mL of 99.5% ethanol. Then, the total volume was adjusted to 25 mL with deionized water. The mixture was incubated in a conical flask with a screw cap at 40 ± 1 °C in a dark room and the degree of oxidation was evaluated by measuring the ferric thiocyanate values. The reaction solution (100 μL) incubated in the linoleic acid model system was mixed with 4.7 mL of 75% ethanol, 0.1 mL of 30% ammonium thiocyanate, and 0.1 mL of 20 mM ferrous chloride solution in 3.5% HCl. After 3 mins, the thiocyanate value was measured by reading the absorbance at 500 nm following color development with FeCl_2_ and thiocyanate at different intervals during the incubation period at 40 ± 1 °C.

### 3.6. Statistical Analysis

All experiments were performed in triplicate (*n* = 3), and an ANOVA test using SPSS 13.0 for Windows (SPSS Inc., Chicago, IL, USA) was used to compare the mean values of each treatment. Significant differences between the means of parameters were determined by using Duncan’s multiple range test (*p* < 0.05).

## 4. Conclusions

In this study, acid soluble collagen (ASC) from scales of croceine croaker (ASC-C) was successfully isolated with yield of 0.37% ± 0.08% (dry weight basis), and characterized as type I collagen on the basis of amino acid analysis and electrophoretic pattern. ASC-C was further hydrolyzed by using double enzyme (trypsin + pepsin), and three antioxidant peptides (ACH-P1, ACH-P2, and ACH-P3) were isolated from the ACH using ultrafiltration, gel chromatography, and RP-HPLC, and their sequences were identified as Glu-Trp-Pro-Ala-Gln (ACH-P1), Phe-Leu-His-Arg-Pro (ACH-P2), and Leu-Met-Gly-Gln-Trp (ACH-P3). Three antioxidant peptides (ACH-P1, ACH-P2, and ACH-P3) showed good radicals scavenging activities and abilities of inhibiting the autooxidation in linoleic acid model system. This research might contribute to a rational basis for the application of the collagen peptides as suitable candidate for exploring functional foods/drugs for the therapy of diseases associated with oxidative stress, or as functional ingredients in food systems to reduce oxidative changes during storage. Further research should be done in order to purify and identify other antioxidative peptides of ACH, and more detailed studies on physiological functions, pharmacological effects and structure-activity relationship of the purified peptides will also be needed.

## Supplementary Files

Supplementary File 1 Supplementary Information (PDF, 60 KB)
