# Supplementary material for: Isolation and Characterization of Collagen and Antioxidant Collagen Peptides from Scales of Croceine Croaker (Pseudosciaena crocea)
Source: Mar Drugs. 2013 Nov 21;11(11):4641–61. doi: 10.3390/md11114641 (PMC3853751; doi:10.3390/md11114641)

Supplementary Information

Figure S1. MALDI-TOF mass spectrogram of ACH-P1 (Gly-Phe-Arg-Gly-Thr-Ile-Gly-Leu-Val-Gly (GFRGTIGLVG)).

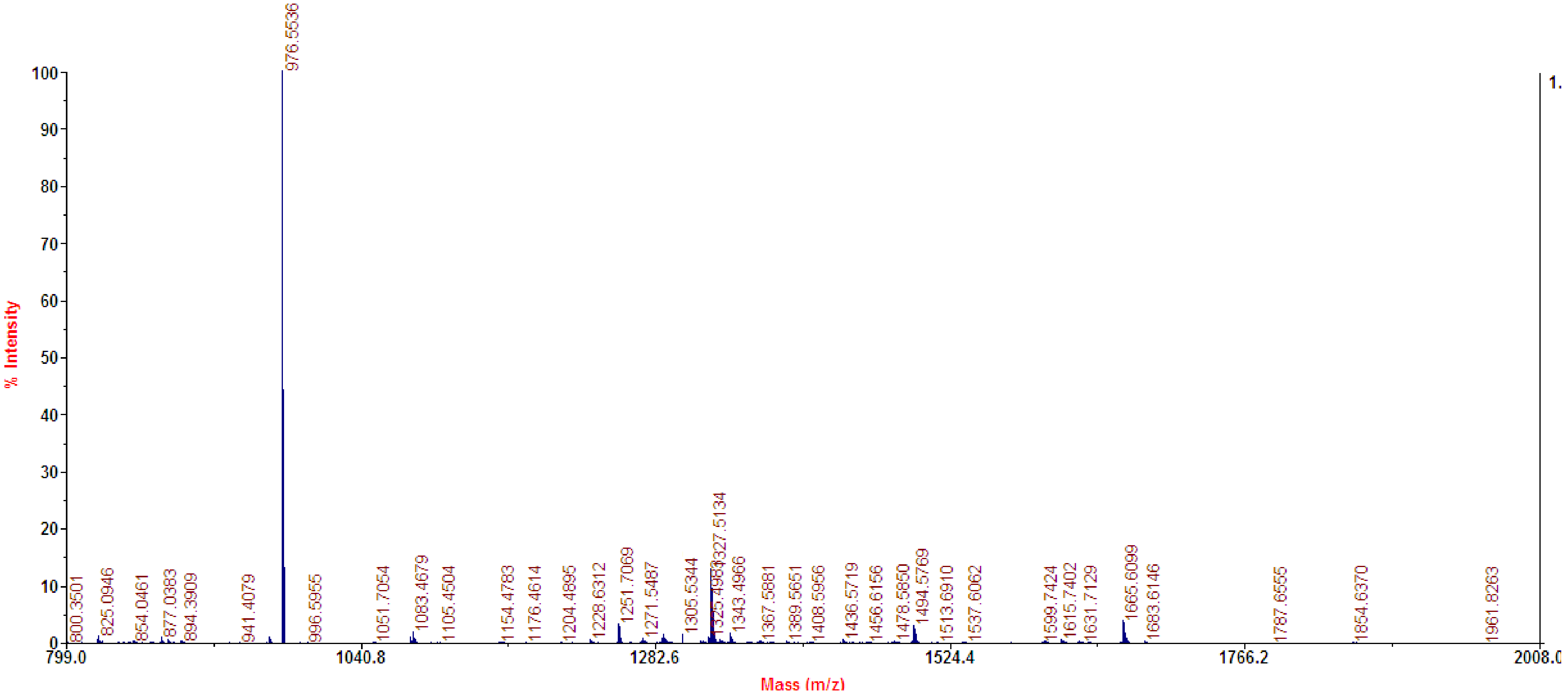

Figure S2. MALDI-TOF mass spectrogram of ACH-P2 (Gly-Pro-Ala-Gly-Pro-Ala-Gly (GPAGPAG)).

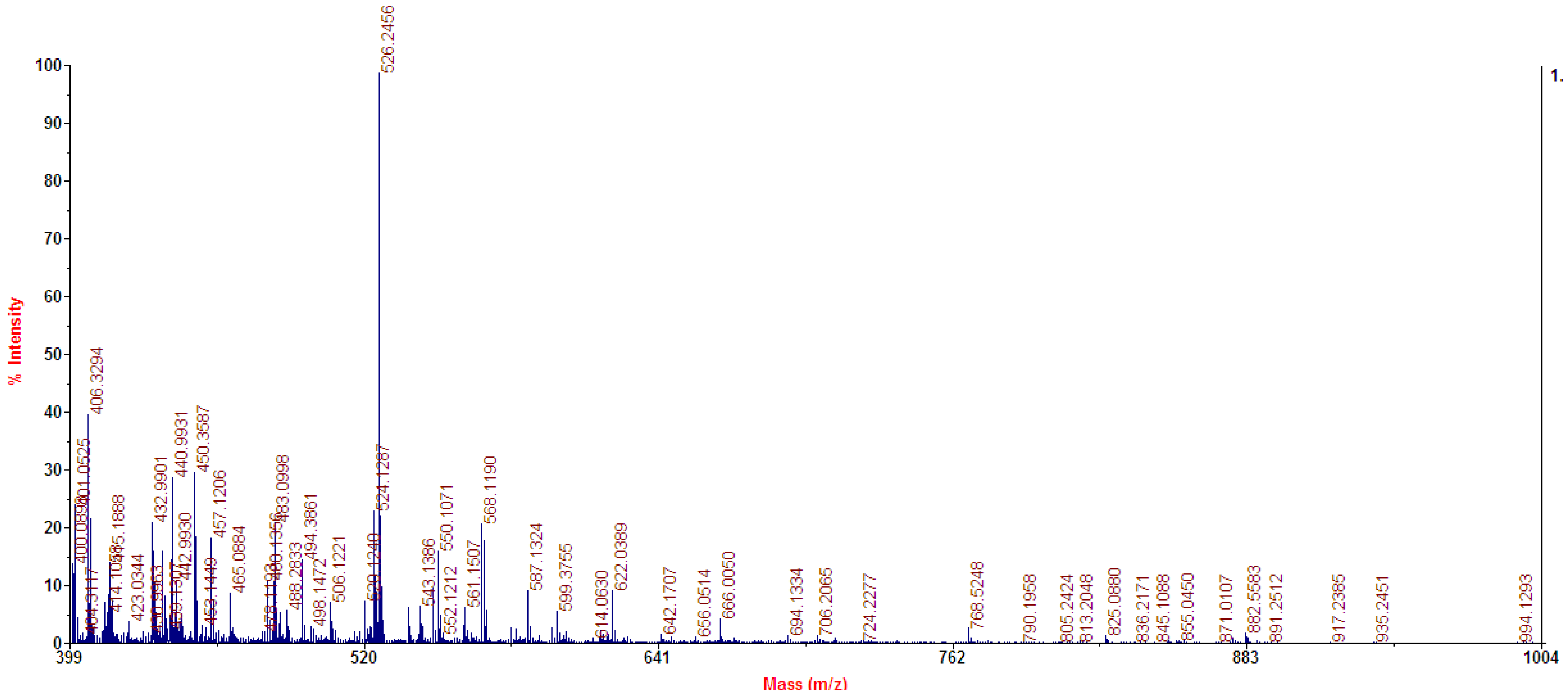

Figure S3. MALDI-TOF mass spectrogram of ACH-P3 (Gly-Phe-Pro-Ser-Gly (GFPSG)).

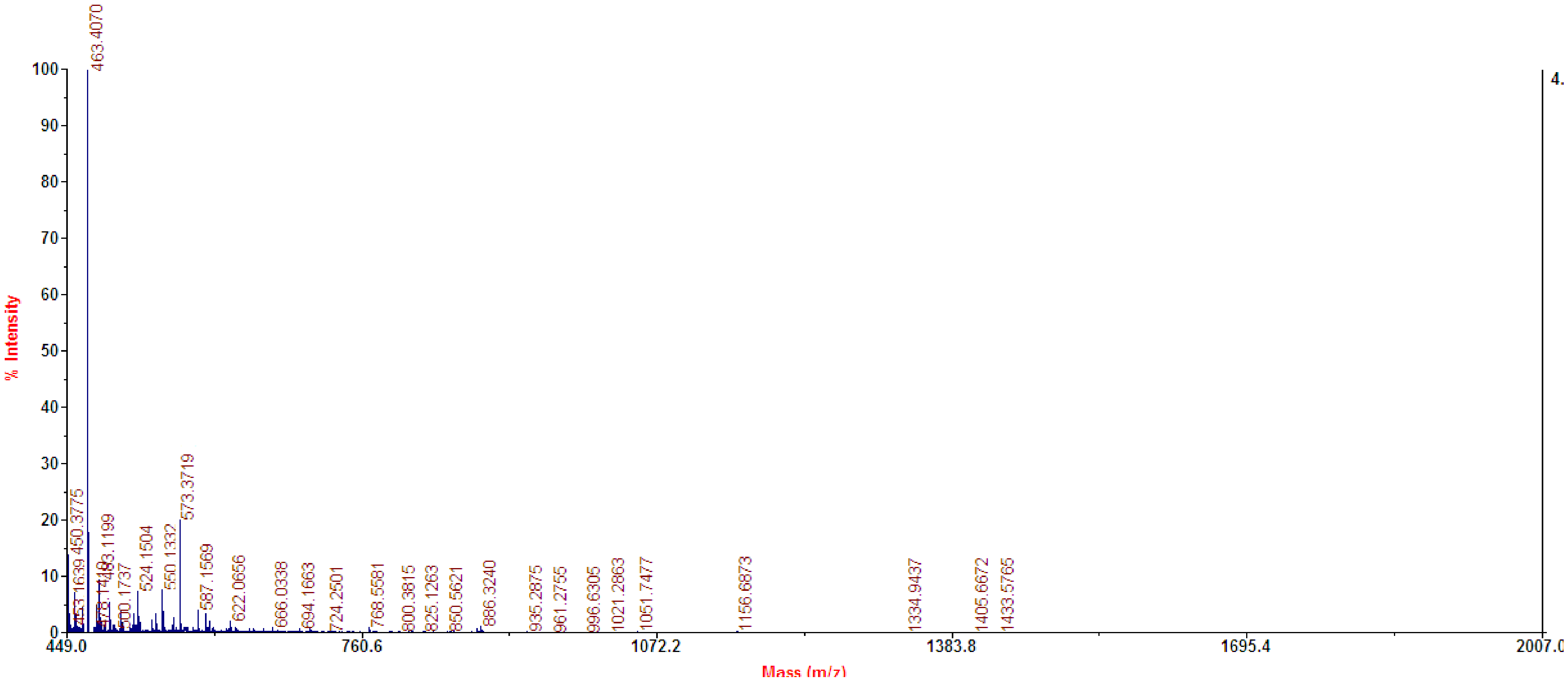

Supplement: Supplementary File 1 — Supplementary Information (PDF, 60 KB) [file marinedrugs-11-04641-s001.pdf]
